# Associations between the innovation climate in vocational universities and students’ innovative behavior: a moderated chain mediation model

**DOI:** 10.3389/fpsyg.2026.1868146

**Published:** 2026-07-17

**Authors:** Weiwei Huang, Xiaoling Xu

**Affiliations:** 1 School of Education, Zhaoqing University, Zhaoqing, China; 2 Guangdong Business and Technology University, Zhaoqing, China

**Keywords:** career calling, innovation climate, innovative behavior, learning engagement, meaning in life, vocational university

## Abstract

**Introduction:**

Cultivating innovative technical talents and stimulating students’ innovative behavior are core objectives of high-quality vocational education. This study aims to examine the internal mechanism linking school innovation climate to innovative behavior among vocational university students.

**Methods:**

A moderated chain mediation model was adopted as the analytical framework. Cross-sectional questionnaire data were gathered from 1,789 vocational university students in Guangdong Province with five validated measurement scales, and regression analysis was performed to test the hypothesized model.

**Results:**

School innovation climate exhibited a significant positive correlation with students’ innovative behavior. Career calling and learning engagement played independent and serial mediating roles, jointly accounting for a total indirect effect of 41.08%. Meaning in life positively moderated three key paths: innovation climate → career calling (*β* = 0.14, *p* < .001), innovation climate → learning engagement (*β* = 0.09, *p* < .001), and innovation climate → innovative behavior (*β* = 0.14, *p* < .001). All correlational relationships became stronger when students reported higher meaning in life.

**Discussion:**

The results clarify the chained mediating and moderating pathways between innovation climate and students’ innovative behavior. This research provides theoretical references and practical intervention strategies for innovation education and the cultivation of innovative skilled talents in vocational colleges.

## 1 Introduction

China’s industrial transformation and the construction of a modern manufacturing system under national industrial upgrading strategies have created massive demand for innovative skilled technical talents [China National Academy of Educational Sciences (NIES), 2026]. From 2015 to 2019, nearly 100 new vocational schools were established nationwide, enrolling more than 2.32 million vocational students [China Global Television Network (CGTN), 2021]. Against the backdrop of technological upgrading, exploring correlational correlates linked to vocational university students’ innovative behavior has become a key research priority for vocational education practice and academia.

Innovative behavior can be understood as the tangible manifestation of an individual’s capacity for innovation within their professional endeavors. According to Scott and Bruce (1994), innovative behavior is a process of value creation supported by innovative ideas, solutions, and applications. Individuals’ innovative behavior drives sustainable development (Kör et al., 2021). Students’ innovative behavior refers to a series of changes in which they generate innovative ideas, try to put them into practice during the learning process, and participate in science and technology activities (Dai et al., 2022). Students’ innovative behavior determines the competitiveness of vocational universities (Liu and Yu, 2021). However, existing empirical research on innovative behavior mainly targets enterprise employees (Khan et al., 2020), while few studies center on vocational school students. Unlike corporate employees whose innovation focuses on organizational profit, vocational students’ innovative activities prioritize technical improvement and process optimization in practical training (Zhu and Xiong, 2020). Therefore, the present study explores the antecedents of vocational students’ innovative behavior, which carries critical practical value for vocational innovation education.

Drawing on social cognitive theory (Bandura, 1986), individual behaviors, internal cognitive-emotional factors, and external environmental factors form a system of reciprocal triadic determinism, rather than being unilaterally constrained by one another. Understanding or anticipating behavior requires viewing individuals and their environment as a set of interdependent variables. Therefore, innovative behavior is closely correlated with the dynamic interplay between individual psychological factors and environmental situational factors. Presently, scholarly investigations extensively employ mediating and moderating effect models, focusing on the mechanism of interaction between situational and individual factors in individuals’ innovative behavior (Zhang et al., 2016).

Schools are an important part of students’ education and lives. Therefore, when studying the factors bearing associations with students’ innovative behavior, the innovation climate of schools is an environmental variable that must be investigated. School climate represents the overall psychological environment shaped by a school’s physical, interpersonal, institutional, and cultural elements (Yang et al., 2024). As a unique subdimension of overall school climate, the school innovation climate refers to the campus atmosphere that recognizes, encourages, and tolerates innovative attempts and trial-and-error practice among faculty and students (Newman et al., 2020). Its core components include peer interaction, teacher support and institutional incentives for innovation, distinct from schools’ routine operational and interpersonal dynamics (Liu and Shi, 2009). While school climate offers a general developmental context for students, innovation climate presents a significant positive correlation with individual innovative behavior.

Previous studies have shown that an innovation climate can be positively associated with innovative behavior via the mediating (Xie, 2015) or moderating effect (Zhao and Zhang, 2020) of other variables. This indicates that the relationship between innovation climate and innovative behavior can be explained from multiple perspectives.

Innovation requires knowledge, which necessitates learning. Technical learning serves as a fundamental representation of vocational education (Zhu and Xiong, 2020). Innovative behavior is a continuous learning-driven process (Xu and Suntrayuth, 2022). Prior work has documented a significant relationship between individuals’ social environment and their career calling (Chen et al., 2023). Additionally, individuals with a strong career calling are more likely to exhibit higher levels of work engagement and an assertive working style, based on social identity theory (Tajfel, 1978). Work engagement plays a crucial role in stimulating work motivation and is a vital mechanism for converting influencing factors into desired attitudes and behaviors (e.g., innovative behavior) as prescribed by organizations (Ćulibrk et al., 2018). Learning engagement is an extension of work engagement in the learning realm (Chen et al., 2016). Thus, career calling and learning engagement can be used as mediating variables in the relationship between innovation climate and innovative behavior. Prior research based on the broaden-and-build theory (Fredrickson, 2004) indicates that individuals with high meaning in life maintain sustained positive affect, which broadens cognitive flexibility and helps them fully capture supportive environmental cues. In line with Schumpeter’s (1939) innovation theory, such expanded cognitive resources further drive individuals to generate and implement innovative technical solutions. Therefore, meaning in life can serve as a moderator in the relationship between innovation climate and other variables.

Based on the aforementioned analysis, it can be inferred that innovation climate, career calling, learning engagement, and meaning in life are pivotal factors influencing innovative behavior. However, previous studies have not integrated these five variables into a comprehensive model. Consequently, the associations linking these factors to innovative behavior remain ambiguous. Drawing upon social cognitive theory, this study incorporates innovation climate, career calling, learning engagement, meaning in life and innovative behavior within a research framework to investigate the associations between innovation climate and innovative behavior.

## 2 Literature review and hypothesis development

### 2.1 Relationship between innovation climate and innovative behavior

School innovation climate can be defined as either objective campus conditions that facilitate innovation or students’ subjective perceptions of institutional innovation support (Xu and Suntrayuth, 2022). According to ecosystem theory (Bronfenbrenner, 1979), schools serve as crucial microsystems for students’ academic and personal development. The innovation climate within schools represents a salient environmental factor that warrants consideration in the study of the factors influencing students’ innovative behavior.

Prior research demonstrated that fostering an environment that endorses and motivates innovation is a viable strategy for promoting innovative practices (Newman et al., 2020). Additionally, the innovation climate displays a significant positive statistical association with innovative behavior (Zhang and Liu, 2017). The psychological underpinnings of innovative behavior within organizations, referred to as the organizational innovation climate (Liu and Shi, 2009), have been shown to display positive associations with such behavior (Xie, 2015). Therefore, cultivating an innovation climate is crucial for developing an ecosystem that fosters the generation, adoption, and implementation of innovative behavior (Xie, 2015). Consequently, we propose the following hypothesis.

*H1*: Innovation climate is positively associated with innovative behavior.

### 2.2 Career calling as a potential mediating variable

Career calling refers to a transcendent inner orientation driven by self-realization and altruistic values (Dik and Duffy, 2009). The concept of career calling refers to an individual’s psychological inclination toward a particular type of work and can function as a significant guiding force aligning their occupation with the broader purpose and meaning in their life. This alignment facilitates the cultivation of greater interests and enables individuals to aid others (Dik and Shimizu, 2019). The dynamic construct of career calling can function as both a cause and an effect variable (Li et al., 2021). As a causal variable, career calling exhibits a significant positive correlation with innovative behavior (Liu and Xu, 2022). The significance of career calling extends to both current students and working adults (Duffy and Dik, 2013), enhancing internal work motivation, and fostering positive attitudes toward work. Additionally, it facilitates the emergence of innovative behavior (Duffy et al., 2015). Career calling serves as a motivating factor for skilled individuals to overcome personal and situational constraints as well as work-related obstacles and challenges while engaging in innovative practices (Zhou et al., 2019).

The factors influencing career calling include not only an individual’s occupational cognition and construction of occupational meaning but also external environmental factors (Shang, 2022). Scholars have conducted research from a dynamic developmental standpoint and discovered that individuals who receive support from their environment are more likely to experience a profound career calling during the initial phases of their careers (Dobrow and Tosti-Kharas, 2012). An innovation climate within an organization can foster a growth-supportive environment for individuals (Zhang and Liu, 2017). Therefore, based on the previous literature, the following hypothesis is proposed:

*H2*: Career calling serves as a mediating factor between innovation climate and innovative behavior.

### 2.3 Learning engagement as a potential mediating variable

Learning engagement refers to students displaying a continuous and positive emotional state during learning activities, characterized by vitality, dedication, and concentration (Schaufeli et al., 2002). Cultivating learning engagement is essential for promoting work engagement in the realm of learning as well as achieving professional knowledge, enhancing skills, and facilitating growth (Chen et al., 2016). Ford’s (1996) creative action model suggests that proficiency and expertise within a specific domain are crucial to promote innovative behavior. This implies that innovation is an ongoing process driven by continuous learning, and fostering active engagement in learning is necessary to express innovative behavior among students (Xu and Suntrayuth, 2022). Technical learning constitutes the fundamental embodiment of vocational education (Zhu and Xiong, 2020). As students in vocational universities cultivate innovative behavior, they must not only reach a certain threshold of knowledge, but also invest time and energy to acquire innovative technologies and ideas.

Simultaneously, the determinants of learning engagement encompass not only personal factors, but also extrinsic factors and their interplay (Yuan et al., 2022). Learning engagement refers to learners’ active and constructive involvement with the external environment and available resources (Wu and Zhang, 2018). It is important to note that there is a significant positive relationship between perceived support from the external environment and students’ engagement in the learning process (Wu et al., 2023). Previous research has indicated that the scholastic reputation of vocational universities bears weaker associations with student learning and development than institutional provision of supportive resources and services (Wang and Wang, 2017). Therefore, we propose the following hypothesis:

*H3*: Learning engagement mediates the relationship between innovation climate and innovative behavior.

### 2.4 Role of career calling and learning engagement in chain mediation

Individuals with career calling demonstrate a strong inclination toward action. These individuals actively engage in practices that align with their career calling (Elangovan et al., 2010). According to identity theory, individuals possessing a career calling are more likely to exhibit higher levels of work engagement and an assertive approach toward their work (Stets and Serpe, 2013). Career calling can foster the perception of work as an integral aspect of one’s life, rather than just an obligation. This perception enhances enthusiasm and commitment toward work, leading to increased creativity and innovation and generating more innovative behaviors (Liu et al., 2021).

Previous studies on pre-service education have identified a significant positive association between career calling and readiness for pre-service learning (Hirschi and Herrmann, 2012). Students who possess a vocational sense of purpose may demonstrate higher aspirations for successful future career outcomes, thereby enhancing their learning engagement during their university years (Shang et al., 2022). Therefore, there is a significant correlation between career calling and learning engagement. Hence, we propose the following hypothesis:

*H4*: Career calling and learning engagement play a chain-mediating role in the relationship between innovation climate and innovative behavior.

### 2.5 Meaning in life as a potential moderating variable

Meaning in life is a subjective experience in which individuals perceive the purpose, value, direction and comprehensibility of their own existence (Martela and Steger, 2016). As a core psychological construct in positive psychology, it consists of two distinct dimensions: the search for meaning and its experiential component (Steger et al., 2006). Scholars regard it as a fundamental human motivation (George and Park, 2017), and extensive evidence has indicated its significant correlation with social behavior (Hu et al., 2023). Meaning in life can function as a protective mechanism against stress. When individuals hold a clear sense of self-worth and life purpose, they are more inclined to regard daily challenges and stress as opportunities for growth rather than obstacles along life’s journey (Park and Baumeister, 2017). Meaning in life plays a critical role in shaping the values of students in contemporary vocational higher education.

Individuals with a high sense of meaning in life can perceive the external environment positively. Positive interaction with the environment can improve cognitive level and psychological resilience (Zhang and Li, 2018), broaden the boundary of cognitive behavior, enhance cognitive flexibility, and stimulate exploratory and innovative behaviors (Li et al., 2022). Meaning in life can expand internal cognitive resources, improve individuals’ ability to absorb external knowledge and career information, while shaping clear learning goals, intellectual curiosity, academic engagement and career calling (Chen, 2021; Zhang et al., 2017). To clarify the interaction mechanism between the external protective factor of innovation climate and the internal protective factor of meaning in life, this study adopts the “protection-protection” model proposed by Liu et al. (2018), which includes two competing hypotheses. The synergistic interaction hypothesis holds that the two factors generate synergistic effects: the positive associative effect is the strongest when both factors are at high levels, and the enhancement effect decreases if only a single factor is sufficient. The antagonistic interaction hypothesis argues that the two factors have a substitution and offset relationship. Due to competition for cognitive psychological resources, when one protective resource is sufficient, the gain from the other will decrease significantly (Liu et al., 2018). This study takes the synergistic interaction hypothesis as the core theory of the moderating effect, based on three rationales: First, social cognitive theory (Bandura, 1986) distinguishes external environmental resources (innovation climate) and internal psychological resources (meaning in life), which do not compete for limited cognitive capacity. Second, the broaden-and-build theory (Fredrickson, 2004) confirms that high meaning in life expands cognitive bandwidth, enabling students to fully utilize campus innovation resources provided by a supportive climate. Third, vocational education empirical evidence (Shang et al., 2022) verifies that internal meaning resources amplify the positive effects of external school support rather than offset them. As an internal cognitive amplifier, meaning in life helps students interpret campus innovation incentives as long-term opportunities to realize occupational value. Thus, meaning in life positively moderates all associative links starting from innovation climate.

Specifically, meaning in life helps students connect the innovation resources in universities with long-term altruistic career values, and strengthens the formation of career calling triggered by environmental support (Dik and Duffy, 2009; Shang et al., 2022). Based on the broaden-and-build theory, higher meaning in life can broaden cognitive bandwidth and buffer the negative emotions caused by academic setbacks, enabling students to fully utilize the resources in the innovation environment and maintain sustained learning engagement (Fredrickson, 2004; Park and Baumeister, 2017). Based on Schumpeter’s innovation theory, meaning in life can stimulate individuals’ autonomous motivation for innovation and improve cognitive flexibility, thereby transforming environmental support for innovation into specific innovative practices (Schumpeter, 1939; Li et al., 2022). However, in the field of vocational education, how the interaction among innovation climate, career calling, learning engagement and meaning in life affects students’ innovative behavior remains unclear. Based on the above comprehensive theoretical deduction, we propose the following hypotheses:

*H5*: Meaning in life moderates the relationship between innovation climate and career calling, learning engagement, and innovative behavior.

### 2.6 Current study

**Figure 1:**
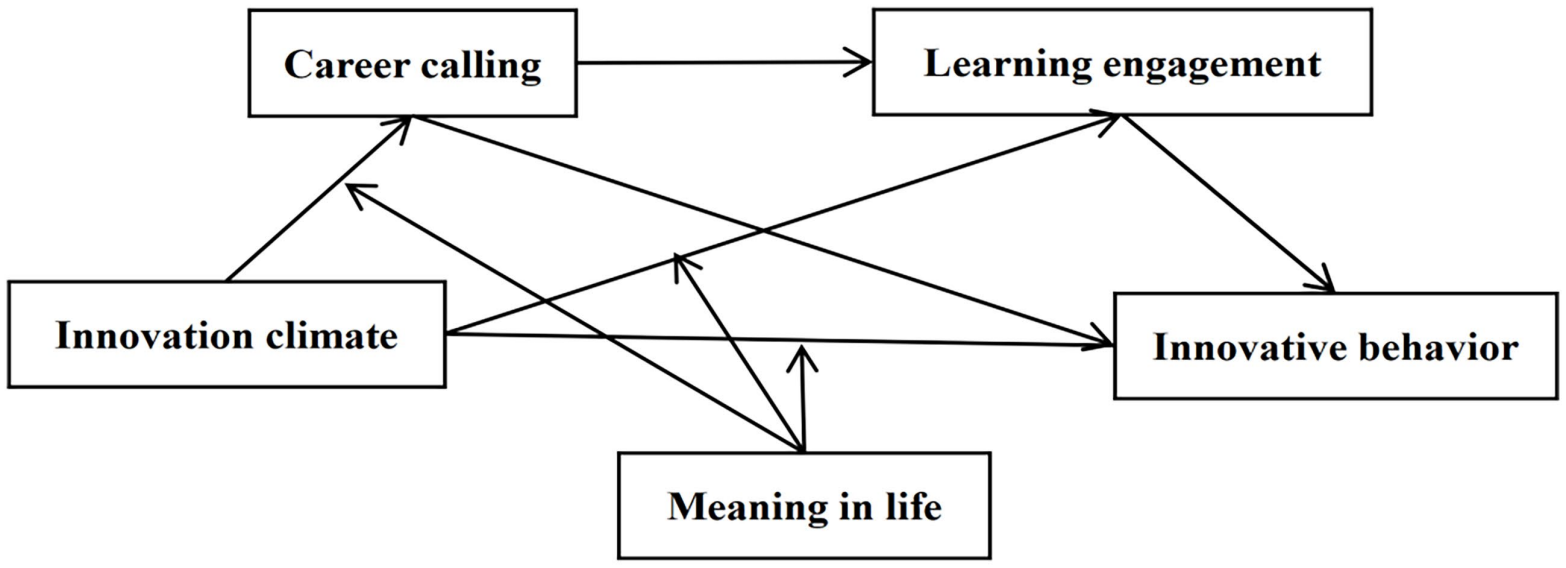
Research hypothesis model.

This study proposes a hypothesized research model (see Figure 1). In this model, innovation climate is treated as the independent variable, innovative behavior as the dependent variable, career calling and learning engagement as mediators, and meaning in life as the moderator. This study examined the statistical association between innovation climate and innovative behavior, as well as the mediating effects of career calling and learning engagement in the association between innovation climate and innovative behavior. Furthermore, it explored the moderating role of meaning in life in the direct and indirect paths between innovation climate and innovative behavior.

## 3 Methods

### 3.1 Sample

This study has been approved by the Ethics Committee of Zhaoqing University (No. 202402). Considering sampling convenience and representativeness, we selected three vocational universities in Guangdong Province, including two comprehensive vocational institutions and one specializing in manufacturing engineering. Data were collected using self-administered electronic questionnaires. Prior to completing the questionnaire, researchers clearly explained the research purpose to participants and informed them that the collected data would be used solely for academic purposes. Participation or withdrawal from the study was entirely voluntary.

A total of 1,981 electronic questionnaires were distributed. After excluding invalid questionnaires (including incomplete responses and incorrect answers to the lie detection questions), 1,789 valid questionnaires were retained, resulting in an effective recovery rate of 90.3%. The mean age of participants was 20.59 ± 1.56 years, comprising 829 (46.34%) male and 960 (53.66%) female students. In terms of grade level distribution, there were 389 (21.74%) freshmen, 411 (22.97%) sophomores, 567 (31.69%) juniors, and 422 (22.21%) seniors. Regarding the origin of the students, individuals from rural areas accounted for 584 (32.64%), those from towns accounted for 640 (35.77%), and those from cities accounted for only a quarter at 565 (31.58%) people.

### 3.2 Measures

#### 3.2.1 Innovation Climate Scale

This study utilized the Innovation Climate Scale proposed by Liu and Shi (2009), which consists of 12 items categorized into three dimensions: classmates, teachers, and school support. An example item is “During my studies, my classmates demonstrate support and assist each other.” A 5-point Likert scale was employed where participants were instructed to rate their level of agreement or disagreement by assigning a numerical value ranging from 1 to 5, representing “1 = strongly disagree” to “5 = strongly agree.” The scale scores ranged from 12 to 60. Higher scores indicated a more significant innovation climate. The overall questionnaire exhibited a satisfactory level of internal consistency with a coefficient of 0.92.

#### 3.2.2 Innovative Behavior Scale

This investigation employed the Innovative Behavior Scale developed by Zhang et al. (2016), which is a self-evaluation scale containing eight questions in a single dimension. An illustrative item reads as follows: “I am always looking for opportunities to improve learning and working methods as well as processes.” A 5-point Likert scale was adopted in which respondents were asked to evaluate their level of agreement/disagreement by providing a quantitative value ranging from 1 to 5, indicating “strongly disagree” or “strongly agree.” The scale scores range from 8 to 40, with higher values indicating a higher level of acknowledgement of innovation among the participants. The overall questionnaire exhibited satisfactory internal consistency, with a coefficient of 0.88.

#### 3.2.3 Learning Engagement Scale

In this study, the Learning Engagement Scale (Fang et al., 2008) was used as a self-evaluation tool. This scale comprises 17 items categorized into three dimensions: vitality, dedication, and focus. For example, one item from the scale is, “I enjoy studying immediately after waking up in the morning.” A 7-point Likert scale was employed, with respondents providing a numerical value ranging from 1 to 7 to indicate the frequency of their behavior. Here, “1” represents “never,” and “7” represents “always.” The scale scores ranged from 17 to 119. The higher the value assigned by respondents, the higher the level of learning engagement. The overall questionnaire demonstrated satisfactory internal consistency, with a coefficient of 0.94.

#### 3.2.4 Career Calling Scale

This study utilized the Career Calling Scale developed by Zhang et al. (2015), which is a self-evaluation scale comprising 11 items divided into three dimensions: altruistic contribution, orientation, and meaningfulness. An illustrative item reads as follows: “I aspire to pursue a career that can benefit others.” A 5-point Likert scale was employed, where respondents were required to assess their level of agreement/disagreement by assigning a quantitative value ranging from 1 to 5, with “1 = strongly disagree” and “5 = strongly agree.” Scores on the scale range from 11 to 55. Higher scores indicated a greater level of career calling experienced by the participants. The internal consistency of the overall questionnaire was deemed satisfactory as evidenced by an internal consistency coefficient of 0.82.

#### 3.2.5 Meaning in Life Scale

The Meaning in Life Scale revised by Wang and Dai (2008) was used in this study. This self-report measure consisted of five items that assessed a single dimension. An illustrative item reads as follows: “I know exactly what gives my life meaning.” A Likert scale with seven response options, ranging from 1 (strongly disagree) to 7 (strongly agree), was employed. The participants were instructed to assign a numerical value to indicate their level of agreement or disagreement. The scores on the scale range from 5 to 35, with a higher score indicating greater perceived meaning in life. The overall questionnaire demonstrated satisfactory internal consistency, with a coefficient of 0.87.

### 3.3 Data analysis

SPSS and AMOS were used for data management and analysis. To unify the rating standard of all scales and avoid statistical deviation caused by inconsistent Likert points (Zhang et al., 2023a,b), we applied the formula “Y = (B-A)*(X-a)/(b-a) + A” (IBM, 2020) to convert the scores of both the Learning Engagement Scale and the Meaning in Life Scale from a 7-level scale to a 5-level scale. Linear transformation only changes the score interval but does not alter the relative rank, correlation and internal structure of the original data, it will not damage the construct validity and criterion validity of the scales (Wu, 2012). For confirmatory factor analysis to assess model fit, AMOS 24.0 was employed. The criteria for evaluating goodness of fit are as follows: *χ*^2^/d*f ≤* 5, RMSEA *≤* 0.08, CFI ≥ 0.90, TLI ≥ 0.90 (Schumacker and Lomax, 2004). Various statistical analyses, including common method bias tests, difference tests, and correlation analyses, were conducted using SPSS 23. Additionally, “PROCESS v3.3” was used for chain mediation analysis and moderated effect analysis. The cross-sectional research design cannot establish a temporal sequence between variables, so all regression coefficients only represent concurrent statistical associations rather than definite causal effects. Future longitudinal designs are required to verify directional causal paths.

## 4 Results

### 4.1 Confirmatory factor analysis and discriminant validity

Before testing the hypotheses, AMOS was employed to conduct a confirmatory factor analysis (CFA) to validate the independence of each variable and discriminant validity. Discriminant validity specifically assesses whether constructs that are theoretically unrelated are correlated. The results of the confirmatory factor analyses (Table 1) revealed that the goodness-of-fit indices for the baseline model (Five-factor model) were as follows: *χ*^2^/d*f* = 2.95, CFI = 0.93, TLI = 0.92, RMSEA = 0.04. The five-factor baseline model exhibited optimal fit indices relative to all collapsed alternative models, confirming adequate discriminant validity among the five core latent constructs.

**Table 1:** Comparison of measurement models.

Model	*χ* ^2^	*df*	*χ*^2^/*df*	CFI	TLI	RMSEA
Five-factor model (baseline model)	3879.25	1,315	2.95	0.93	0.92	0.04
Four-factor model^a^	8976.12	1,319	6.81	0.88	0.88	0.05
Three-factor model^b^	11224.18	1,322	8.49	0.85	0.84	0.05
Two-factor model^c^	15914.86	1,324	12.02	0.78	0.77	0.07
One-factor model^d^	23997.02	1,325	18.11	0.66	0.64	0.08

CFI, comparative fit index; TLI, Tucker-Lewis’s index; RMSEA, root mean square error of approximation.

a This model combines innovative behavior and meaning in life into one factor.

b This model combines innovative behavior, meaning in life, and career calling into a single factor.

c This model combines innovative behavior, meaning in life, career calling, and innovation climate into a single factor.

d This model combines innovative behavior, meaning in life, career calling, innovation climate, and learning engagement into a single factor.

### 4.2 Common method bias

Harman’s single-factor test (Harman, 1976) was used to assess the presence of a common method bias. The findings revealed that the first unrotated principal component explained only 32.76% of the total variance, which fell below the 40% critical threshold, suggesting no severe common method bias.

### 4.3 Difference test and correlation analysis

The results of the difference tests demonstrated that origin displayed significant differences in participants’ scores for innovative behavior (*F* = 15.69, *p* < 0.001), learning engagement (*F* = 3.78, *p* < 0.05), innovation climate (*F* = 7.34, *p* < 0.001), career calling (*F* = 13.27, *p* < 0.001), and meaning in life (*F* = 3.18, *p* < 0.05). Furthermore, gender was found to display significant associations with participants’ scores in the innovation climate (*t* = −3.26, *p* < 0.001) and career calling (*t* = −2.98, *p* < 0.001). Additionally, grade significantly affected participants’ scores on innovative behavior (*F* = 3.18, *p* < 0.05), learning engagement (*F* = 5.95, *p* < 0.001), innovation climate (*F* = 4.76, *p* < 0.001), and career calling (*F* = 3.30, *p* < 0.05).

The premise of model testing is that there is a significant correlation between the main variables. Therefore, we conducted a bivariate correlation analysis to examine the relationships among the variables. The findings from the analysis, presented in Table 2, demonstrate a significant positive correlation between age, learning engagement, and innovation climate. Additionally, significant positive correlations are observed between the primary variables of innovation climate, learning engagement, innovative behavior, career calling, and meaning in life. Therefore, it is necessary to further investigate the relationships between innovative behavior, meaning in life, learning engagement, innovation climate, and career calling. Grade, age, gender, and place of origin were considered as control variables during model testing.

**Table 2:** Means, standard deviations, correlations for the main study variables (*N* = 1789).

Thematic content	*M*	*SD*	1. Age	2. Innovative behavior	3. Innovation climate	4. Learning engagement	5. Career calling
1. Age	20.57	1.57	–				
2. Innovative behavior	26.96	6.45	0.03	–			
3. Innovation climate	42.08	9.35	0.04^*^	0.56^***^	–		
4. Learning engagement	49.58	11.68	0.07^**^	0.54^***^	0.46^***^	–	
5. Career calling	37.88	6.70	0.02	0.54^***^	0.50^***^	0.59^***^	–
6. Meaning in life	14.43	3.39	0.02	0.33^***^	0.46^***^	0.41^***^	0.38^***^

*p < 0.05, **p < 0.01, ***p < 0.001.

### 4.4 The results of chain mediation effect

The independent variable in this study was innovation climate, while the dependent variable was innovative behavior. Additionally, career calling and learning engagement were considered as mediating variables, and grade, age, gender, and place of origin were included as control variables to examine the chain-mediating effect. The chain-mediating effect was tested using the SPSS macro program PROCESS V3.3 (Model 6), developed by Hayes (2022). The findings (Table 3) indicated that there was a positive correlation between the innovation climate and innovative behavior when mediating variables were not present (*β* = 0.56, *t* = 33.95, *p* < 0.001), thereby supporting H1.

**Table 3:** The results of the chain mediation model (*N* = 1789).

Dependent variables	Independent variables	*β*	SE	*t*	95%CI	*p*
Innovative behavior	Innovation climate	0.56	0.02	33.95	[0.53, 0.59]	<0.001
	Gender	0.01	0.03	0.38	[−0.05,0.08]	0.71
	Grade 1	−0.04	0.06	−0.63	[−0.17,0.08]	0.53
	Grade 2	−0.06	0.06	−1.13	[−0.17,0.05]	0.26
	Grade 3	−0.02	0.05	−0.28	[−0.12,0.09]	0.78
	Origin 1	−0.10	0.04	−2.26	[−0.18,-0.01]	0.02
	Origin 2	−0.18	0.04	−4.14	[−0.26,-0.09]	<0.001
Career calling	Innovation climate	0.50	0.02	28.77	[0.46, 0.53]	<0.001
	Gender	−0.04	0.03	−1.21	[−0.11,0.03]	0.23
	Grade 1	−0.05	0.07	−0.75	[−0.18,0.08]	0.45
	Grade 2	0.01	0.06	0.25	[−0.10,0.13]	0.80
	Grade 3	0.04	0.06	0.77	[−0.09, 0.16]	0.44
	Origin 1	0.00	0.04	0.00	[−0.09,0.09]	1.00
	Origin 2	−0.15	0.05	−3.31	[−0.24,-0.06]	<0.001
Learning engagement	Innovation climate	0.22	0.02	12.12	[0.18, 0.25]	<0.001
	Career calling	0.48	0.02	26.62	[0.45, 0.52]	<0.001
	Gender	0.11	0.03	3.43	[0.05,0.17]	<0.001
	Grade 1	−0.10	0.06	−1.66	[−0.22,0.02]	0.10
	Grade 2	−0.12	0.05	−2.36	[−0.23,-0.02]	<0.05
	Grade 3	−0.04	0.05	−0.76	[−0.14,0.06]	0.45
	Origin 1	−0.04	0.04	−0.97	[−0.12,0.04]	0.33
	Origin 2	0.01	0.04	0.31	[−0.07,0.09]	0.76
Innovative behavior	Innovation climate	0.33	0.02	18.92	[0.30, 0.37]	<0.001
	Career calling	0.22	0.02	11.42	[0.18, 0.26]	<0.001
	Learning engagement	0.26	0.02	13.66	[0.22, 0.29]	<0.001
	Gender	0.00	0.03	−0.03	[−0.06,0.06]	0.98
	Grade 1	0.00	0.06	0.05	[−0.11,0.12]	0.96
	Grade 2	−0.04	0.05	−0.72	[−0.13,0.06]	0.47
	Grade 3	−0.02	0.05	−0.41	[−0.12,0.08]	0.68
	Origin 1	−0.09	0.04	−2.25	[−0.16,-0.01]	<0.05
	Origin 2	−0.13	0.04	−3.35	[−0.21,-0.05]	<0.001

Coefficients are standardized; bootstrap samples = 5,000; CI, confidence interval; grade and origin are multi-category non-interval categorical variables. If directly entered into regression models, they will create erroneous linear assumptions; thus, dummy coding is required to accurately control baseline group differences and yield unbiased mediation and moderation parameters.

The results of the mediation analysis indicated that the innovation climate shared a significant positive statistical association with both career calling (*β* = 0.50, *t* = 28.77, *p* < 0.001) and learning engagement (*β* = 0.22, *t* = 12.12, *p* < 0.001) (Table 4). Additionally, career calling was found to be positively associated with both learning engagement (*β* = 0.48, *t* = 26.62, *p* < 0.001) and innovative behavior (*β* = 0.22, *t* = 11.42, *p* < 0.001). Furthermore, there was a positive relationship between learning engagement and innovative behavior (*β* = 0.26, *t* = 13.66, *p* < 0.001).

**Table 4:** Indirect effects, direct effect, and total effect (*N* = 1789).

Types of effects	Paths	Effect	Boot	Boot	Boot	Percentage
			SE	LLCI	ULCI	
Total effect	Innovation climate → innovative behavior	0.56	0.02	0.53	0.59	
Direct effect	Innovation climate → innovative behavior	0.33	0.02	0.30	0.37	58.92%
Indirect effects	Total indirect effect	0.23	0.02	0.20	0.26	41.08%
	Ind1 Innovation climate → career calling → innovative behavior	0.11	0.01	0.08	0.14	19.64%
	Ind2 Innovation climate → learning engagement → innovative behavior	0.06	0.01	0.04	0.07	10.71%
	Ind3 Innovation climate → career calling → learning engagement → innovative behavior	0.06	0.01	0.05	0.08	10.71%
	Ind1 minus Ind2	0.05	0.02	0.02	0.09	
	Ind1 minus Ind3	0.05	0.02	0.01	0.08	
	Ind2 minus Ind3	−0.01	0.01	−0.02	0.01	

Coefficients are standardized; Bootstrap samples = 5,000; Boot LLCI, the bootstrapping lower limit confidence interval; Boot ULCI, the bootstrapping upper limit confidence interval.

The results presented in Table 4 indicate that the path “Innovation climate → innovative behavior” exhibited a statistically significant value (effect = 0.33, *SE* = 0.02, 95% CI = [0.30, 0.37]), with an effect ratio of 58.92%. Additionally, there were three significant indirect paths: “Innovation climate → career calling → innovative behavior,” “innovation climate → learning engagement → innovative behavior,” and “innovation climate → career calling → learning engagement → innovative behavior.” The path “Innovation climate → career calling → innovative behavior” had a significant effect (effect = 0.11, *SE* = 0.01, 95% CI = [0.08, 0.14]) and an effect ratio of 19.64%, providing support for H2. Similarly, the path “Innovation climate → learning engagement → innovative behavior” demonstrated a significant effect (effect = 0.06, *SE* = 0.01, 95% CI = [0.04, 0.07]) and an effect ratio of 10.71%, confirming H3. Furthermore, the path “Innovation climate → career calling → learning engagement → innovative behavior” also exhibited a significant effect (effect = 0.06, *SE* = 0.01, 95% CI = [0.05, 0.08]) and an effect ratio of 10.71%, supporting H4. The independent indirect pathway through career calling alone (Ind1) carried the largest standardized indirect effect (*β* = 0.11), accounting for 19.64% of the total statistical association between innovation climate and innovative behavior (vs. Ind2: *Δ* = 0.05, 95% CI [0.02, 0.09]; vs. Ind3: Δ = 0.05, 95% CI [0.01, 0.08]). Notably, the two paths involving learning engagement (Ind2 and Ind3) did not differ significantly from each other (Δ = −0.01, 95% CI [−0.02, 0.01]), suggesting that learning engagement’s contribution to innovative behavior is comparable whether it operates independently or sequentially after career calling.

### 4.5 Moderated mediation model

The moderated mediation model was analyzed using the SPSS macro program PROCESS V 3.3 (Model 85) developed by Hayes (2022). The results presented in Table 5 indicated that the interaction between innovation climate and meaning in life was significantly and positively associated with career calling (*β* = 0.14, *t* = 10.35, *p* < 0.001), learning engagement (*β* = 0.09, *t* = 7.40, *p* < 0.001), and innovative behavior (*β* = 0.14, *t* = 11.56, *p* < 0.001), supporting H5.

**Table 5:** Results of the moderated mediator model test (*N* = 1789).

Dependent variables	Independent variables	*β*	SE	*t*	95%CI	*p*
Career calling	Innovation climate	0.45	0.02	23.62	[0.41,0.49]	<0.001
	Meaning in life	0.17	0.02	9.32	[0.14,0.21]	<0.001
	Innovation climate × meaning in life	0.14	0.01	10.35	[0.11,0.17]	<0.001
	Gender	−0.06	0.03	−1.86	[−0.13, 0.00]	0.06
	Grade 1	−0.04	0.06	−0.7	[−0.17, 0.08]	0.49
	Grade 2	0.03	0.06	0.52	[−0.08, 0.14]	0.60
	Grade 3	0.05	0.06	0.94	[−0.06, 0.16]	0.35
	Origin 1	0	0.04	0.07	[−0.08, 0.09]	0.94
	Origin 2	−0.12	0.04	−2.86	[−0.21, −0.04]	<0.05
Learning engagement	Innovation climate	0.20	0.02	10.22	[0.16,0.24]	<0.001
	Career calling	0.42	0.02	23.00	[0.38,0.46]	<0.001
	Meaning in life	0.16	0.02	9.13	[0.12,0.19]	<0.001
	Innovation climate × meaning in life	0.09	0.01	7.40	[0.07,0.12]	<0.001
	Gender	0.09	0.03	2.92	[0.03, 0.15]	<0.05
	Grade 1	−0.1	0.06	−1.7	[−0.22, 0.02]	0.09
	Grade 2	−0.11	0.05	−2.22	[−0.21, −0.01]	<0.05
	Grade 3	−0.03	0.05	−0.66	[−0.13, 0.07]	0.51
	Origin 1	−0.04	0.04	−1	[−0.12,0.04]	0.32
	Origin 2	0.02	0.04	0.55	[−0.06,0.10]	0.58
Innovative behavior	Innovation climate	0.40	0.02	21.32	[0.36,0.43]	<0.001
	Career calling	0.20	0.02	10.27	[0.16,0.23]	<0.001
	Learning Engagement	0.23	0.02	12.11	[0.19,0.26]	<0.001
	Meaning in life	−0.02	0.02	−1.05	[−0.05,0.02]	0.29
	Innovation climate × meaning in life	0.14	0.01	11.56	[0.12,0.16]	<0.001
	Gender	0.01	0.03	−0.32	[−0.07,0.05]	0.75
	Grade 1	0	0.06	0.06	[−0.11, 0.11]	0.95
	Grade 2	−0.02	0.05	−0.47	[−0.12,0.07]	0.64
	Grade 3	0	0.05	−0.08	[−0.10, 0.09]	0.94
	Origin 1	−0.07	0.04	−2	[−0.15, 0.00]	<0.05
	Origin 2	−0.12	0.04	−3.06	[−0.19,-0.04]	<0.05

Coefficients are standardized; bootstrap samples = 5,000; CI, confidence interval; grade and origin are multi-category non-interval categorical variables. If directly entered into regression models, they will create erroneous linear assumptions; thus, dummy coding is required to accurately control baseline group differences and yield unbiased mediation and moderation parameters.

**Figure 2:**
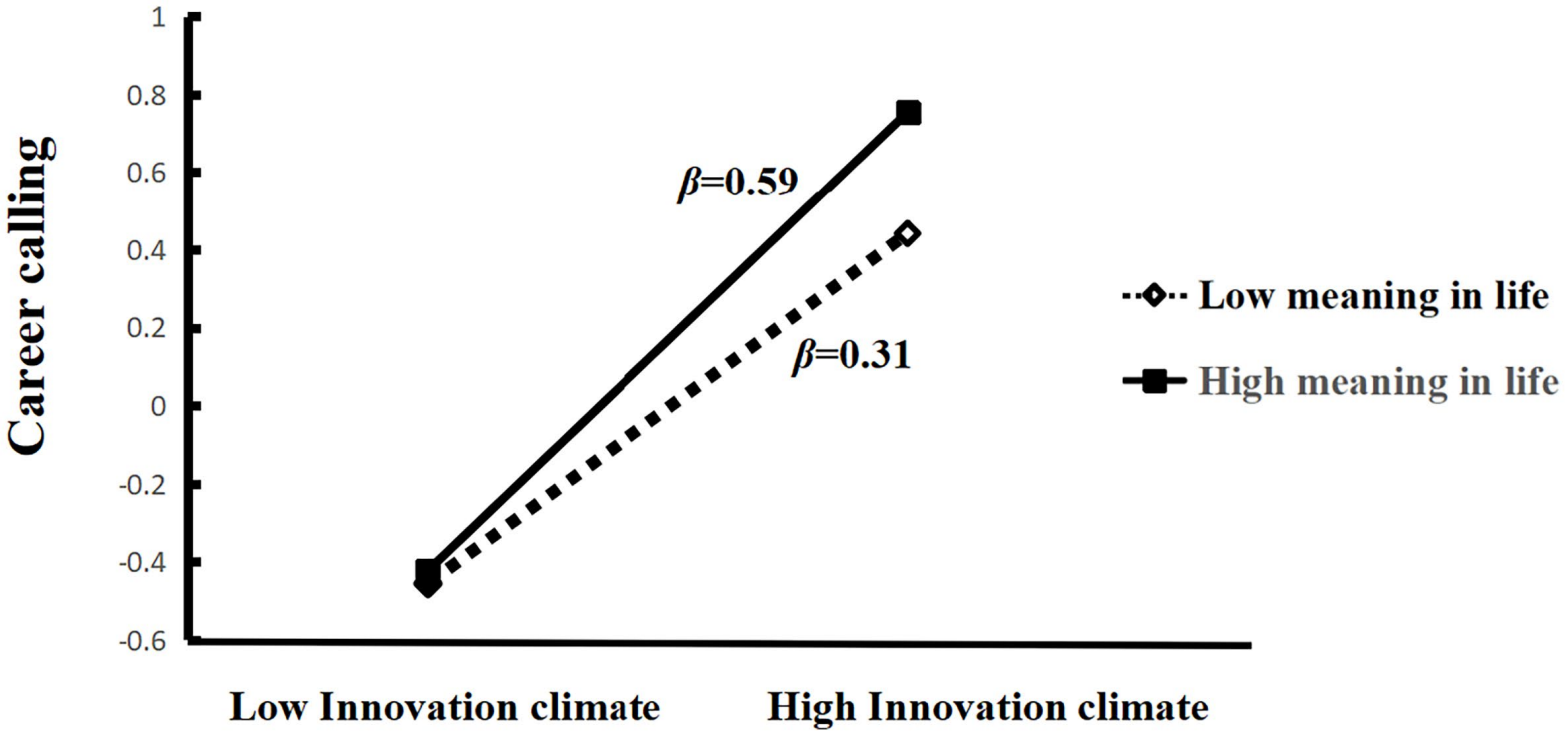
Moderating effect of meaning in life on innovation climate and career calling.

To intuitively illustrate the moderating effect of meaning in life, we divided the score of meaning in life into two groups for simple slope analysis (Aiken et al., 1991) using the criterion of plus or minus one standard deviation. The results (Figure 2) indicated that in the group with high meaning in life (+1 *SD*), innovation climate had a stronger associative effect on career calling (*β* = 0.59, *SE* = 0.03, *p* < 0.001). In the group with low meaning in life (−1 *SD*), the strength of the statistical association between innovation climate and career calling was weaker (*β* = 0.31, *SE* = 0.02, *p* < 0.001).

**Figure 3:**
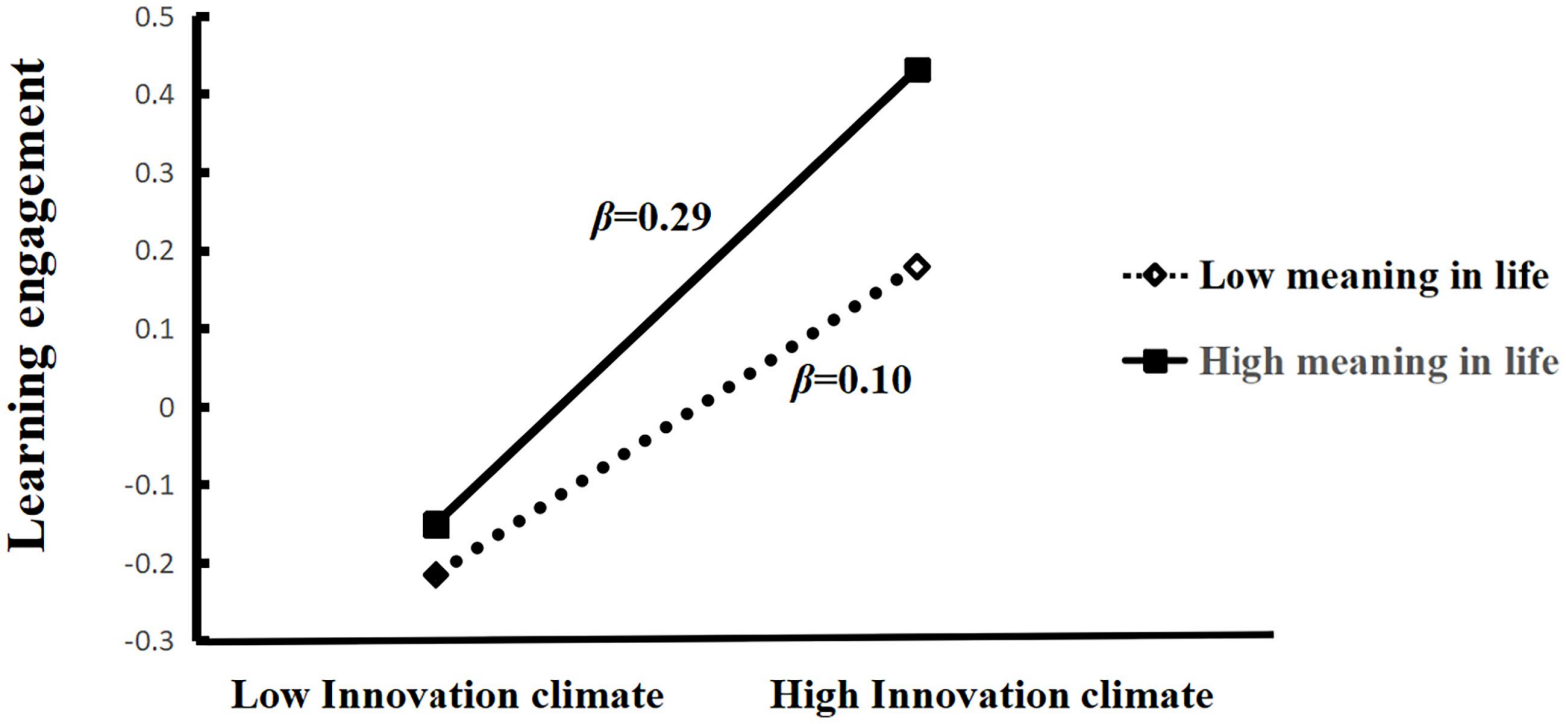
Moderating effect of meaning in life on innovation climate and learning engagement.

The findings (Figure 3) revealed that in the high meaning in life group (+1 *SD*), the associative effect of innovation climate on learning engagement was stronger (*β* = 0.29, *SE* = 0.03, *p* < 0.001). Conversely, in the low meaning in life group (−1 *SD*), the associative effect of innovation climate on learning engagement was weaker (*β* = 0.10, *SE* = 0.02, *p* < 0.001).

**Figure 4:**
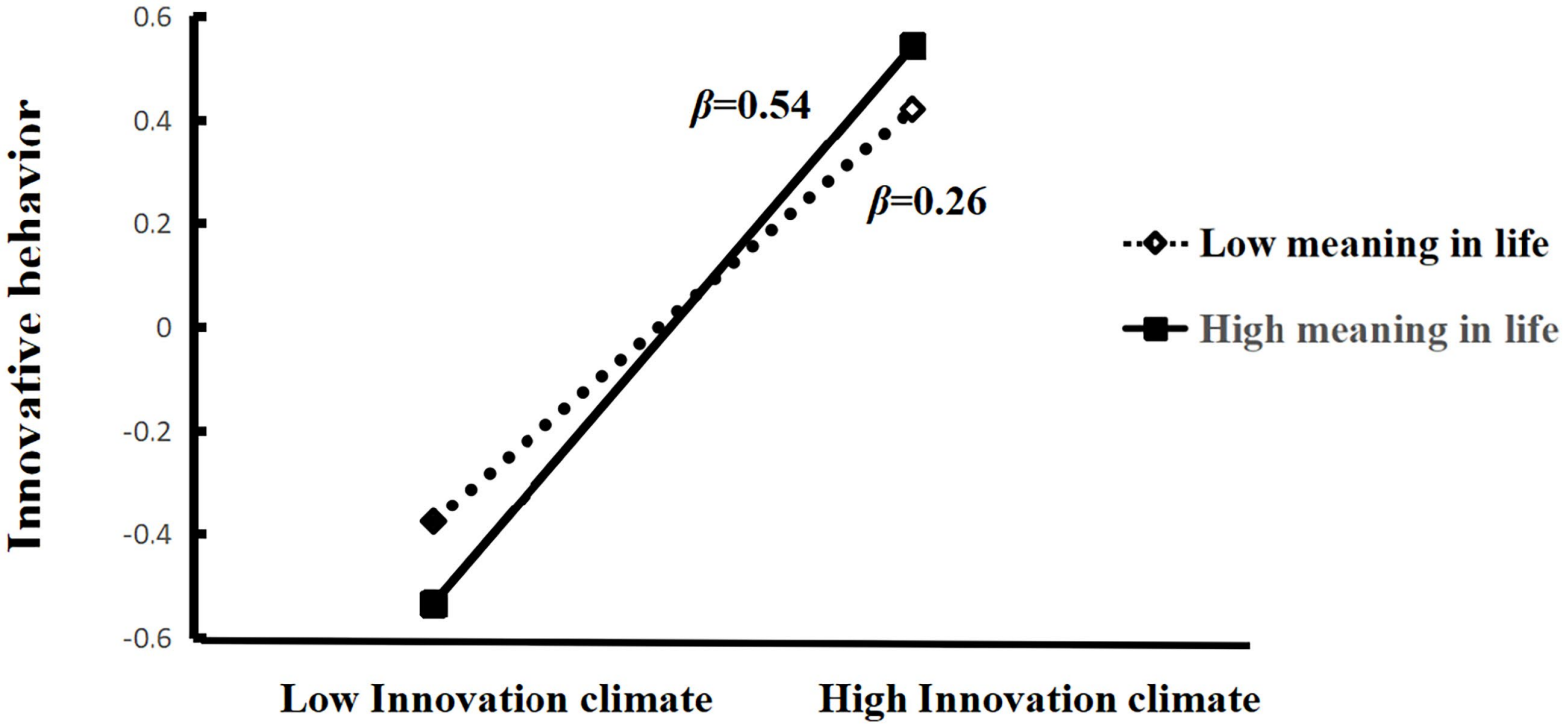
Moderating effect of meaning in life on innovation climate and innovative behavior.

The results (Figure 4) indicated that in the group with high meaning in life (+1 *SD*), innovation climate had a stronger associative effect on innovative behavior (*β* = 0.54, *SE* = 0.03, *p* < 0.001), and in the group with low meaning in life (−1 *SD*), innovation climate had a weaker associative effect on innovative behavior (*β* = 0.26, *SE* = 0.02, *p* < 0.001). In summary, meaning in life positively moderates the associative effect of innovation climate on career calling, learning engagement, and innovative behavior.

## 5 Discussion

### 5.1 The relationship between innovation climate and innovative behavior

Consistent with Hypothesis H1, school innovation climate shows a significant positive concurrent statistical association with vocational university students’ innovative behavior, which aligns with prior research findings based on corporate employee and undergraduate samples (Newman et al., 2020; Xu and Suntrayuth, 2022). Nevertheless, this study extends existing literature to skill-oriented vocational education populations. Unlike undergraduates who focus on theoretical creation, vocational university students’ innovative activities center on technical optimization (Zhu and Xiong, 2020). Peer communication, teachers’ technical guidance, and institutional incentive mechanisms for school maker studios serve as core drivers that facilitate their innovative practices (Thurlings et al., 2015). A failure-tolerant campus innovation atmosphere reduces students’ fear of trial and error, enabling them to translate environmental support into concrete technical improvement behaviors (Herting, 2000).

### 5.2 Mediating effect

The results of the mediation analysis indicate that innovation climate is significantly and indirectly related to innovative behavior through both independent mediation pathways—via career calling and learning engagement—and a chained mediation pathway involving career calling and learning engagement, supporting hypotheses H2, H3, and H4. This finding aligns with Zhou et al. (2019), who confirmed that career calling plays a significant independent mediating role between environmental support and innovation performance. Bootstrap comparisons of indirect effect sizes revealed that the independent mediation effect of career calling (Ind1) was significantly larger than the two paths associated with learning engagement (Ind2 and Ind3). From a theoretical perspective, career calling represents a stable, long-term occupational identity rooted in altruistic values (Hirschi and Herrmann, 2012), whereas learning engagement is a transient, situational response to environmental support (Schaufeli et al., 2002). Thus, when vocational university students perceive a supportive campus innovation environment, their sense of career calling is strengthened. This enduring identity further stimulates learning engagement and ultimately promotes innovative practices, explaining why the career calling pathway accounts for the dominant indirect effect (Zhou et al., 2019).

The significant chain mediation effect (innovation climate → career calling → learning engagement → innovative behavior) suggests that students with strong career calling exhibit greater learning engagement, consistent with prior research (Yuan et al., 2022; Thurlings et al., 2015). Ford (1996) posits that the creative action model emphasizes the importance of expertise and proficiency in specific domains for stimulating innovative behavior. Students with strong career calling tend to identify strongly with their chosen profession and are more likely to participate actively in relevant learning and practical activities during pre-service training to achieve future career goals, prepare for professional development, and continuously enhance their technical competence (Hirschi and Herrmann, 2012).

### 5.3 Moderating effect of meaning in life

This study finds that meaning in life amplifies the strength of the statistical association between innovation climate and career calling, learning engagement, and innovative behavior, fully supporting hypothesis H5. This finding aligns with the enhancement interaction hypothesis proposed by the Conservation of Resources (COR) model (Liu et al., 2018), which posits that two complementary protective factors produce mutually reinforcing effects. An innovation climate functions as an external environmental protective factor, providing individuals with innovation support from peers, teachers, and institutional systems; meanwhile, meaning in life serves as an internal psychological resource, offering stable value anchors, cognitive flexibility, and resilience to stress. These two protective factors operate synergistically rather than competitively, explaining why meaning in life positively moderates each pathway from innovation climate to subsequent psychological and behavioral outcomes. Drawing on Bandura’s social cognitive theory (1986), external environmental stimuli cannot directly determine individuals’ vocational identity, learning states, or innovative behaviors; instead, students rely on their internal meaning-making systems to interpret and absorb cues from the environment. Meaning in life acts as a unifying cognitive filter, reshaping how students perceive school-based innovation resources, thereby amplifying the positive associative strength of the innovation climate across all stages of the model.

Career calling is a transcendent sense of purpose rooted in altruistic contribution (Dik and Duffy, 2009), emerging from both external environments and individual construction of professional meaning. High levels of meaning in life foster mature value judgment systems (Martela and Steger, 2016): when exposed to a campus environment that embraces trial-and-error and encourages innovation, students with high meaning in life can link environmental support with the long-term societal value of technical careers, interpreting campus innovation incentives as institutional endorsement within the tech industry—thus amplifying the positive effect of the innovation climate on career calling. In contrast, students with low meaning in life lack stable value anchors and struggle to integrate campus innovation resources into their personal career aspirations. This inference resonates with Zhang et al. (2023a,b), who concluded that meaning in life positively moderates the relationship between environmental support and career calling. Furthermore, meaning in life satisfies individuals’ intrinsic needs for goal attainment and belonging, transforming external innovation policies into endogenous career motivation and deepening the internalization of career calling (Duffy et al., 2015). Simple slope analyses confirm this mechanism: under low meaning in life, the statistical association between innovation climate and career calling equals *β* = 0.31 at low levels of meaning in life, whereas under high meaning in life, this effect increases to *β* = 0.59.

Learning engagement refers to a sustained, positive state of learning driven by both environmental conditions and vocational identity, encompassing vigor, dedication, and absorption (Schaufeli et al., 2002). Meaning in life moderates the associations of the innovation climate with learning engagement through the broaden-and-build theory and stress-buffering mechanisms. High meaning in life fosters positive emotions, broadens cognitive scope, and enables students to fully utilize innovation resources such as competitions and practical training, thus deepening their academic commitment (Fredrickson, 2004). In contrast, individuals with low meaning in life experience cognitive constraints, making it difficult for them to convert environmental support into meaningful learning (Li et al., 2022). Skill acquisition involves repeated trial and error, and meaning in life buffers the stress associated with setbacks, helping students view challenges as growth opportunities. The failure-tolerant nature of the innovation climate further amplifies this protective effect (Park and Baumeister, 2017). Simple slope analysis confirms this: at low levels of meaning in life, the correlational coefficient stands at *β* = 0.10, increasing to *β* = 0.29 at high levels of meaning in life.

Innovative behavior encompasses the entire process through which students generate creative ideas and implement improvements in technology and processes during their academic studies (Dai et al., 2022). The moderating role of meaning in life can be explained by integrating Schumpeter’s theory of innovation and cognitive flexibility theory. Schumpeter (1939) argued that innovation stems from endogenous breakthrough motivation. Students with high meaning in life transcend utilitarian incentives, leveraging campus innovation platforms to realize social value and proactively engage in technological advancement; in contrast, students with low meaning in life merely pursue awards and monetary rewards, showing weak intrinsic motivation for innovation. Meaning in life also expands cognitive resources and enhances mental flexibility, helping students break free from conventional technical thinking and reorganize their professional knowledge (Zhang and Li, 2018), thereby transforming environmental support into actual innovative actions linked to an innovation-oriented atmosphere. Simple slope analysis revealed that the correlational coefficient was *β* = 0.26 at low levels of meaning in life, significantly increasing to *β* = 0.54 at high levels.

Bandura’s (1986) triadic reciprocal determinism implies mutual interconnections among individuals’ psychological states, environmental conditions, and behavioral expressions, rather than a unidirectional causal chain from environment to mind to behavior. Although our cross-sectional data cannot verify bidirectional feedback loops over time, existing research provides theoretical clues for simultaneous bidirectional associations: higher self-reported innovative behaviors are linked with stronger occupational calling (Liu et al., 2021); sustained innovation exploration correlates with persistent learning engagement (Yuan et al., 2022); students’ collective innovative behaviors send positive signals that encourage schools to improve innovation initiatives and continuously enhance the campus innovation climate (Fan and Li, 2026); frequent innovative practices are associated with a greater sense of life meaning (Li et al., 2022). The cross-sectional data used in this study cannot capture such dynamic bidirectional causal cycles. Future longitudinal tracking studies are needed to clarify temporally ordered patterns of bidirectional relationships.

## 6 Implications

This moderated chain mediation model offers practical strategies for vocational university administrators, teachers and enterprise mentors across curriculum reform, psychological intervention and school-enterprise cooperation. First, the independent mediating effect of career calling is significantly higher than the independent mediating effect of learning engagement and the chain mediating effect. This correlation pattern suggests that when advancing innovation-oriented education reform, special modules can be designed to explore programs for strengthening the activation of students’ career calling, such as introducing narratives of industry role models and organizing seminars on technical public welfare cases. However, given the cross-cultural measurement sensitivity of career calling and the regional limitations of the research samples in this study, the design and effectiveness of such modules require repeated verification across multiple institutional contexts. Second, there is no significant difference between the effect of the independent mediating path of learning engagement and that of the chain mediating path, indicating that learning engagement may function through two mechanisms: direct skill accumulation and indirect identity construction. Based on this correlation pattern, education administrators can start by optimizing classroom interaction design and improving the process-oriented evaluation system, and then explore strategies to mobilize students’ engagement enthusiasm. Pilot studies can comparatively examine the relative effects of direct technical training versus learning programs integrating career calling reflection on improving learning engagement and subsequent innovative behaviors, so as to identify the optimal intervention path. Third, the interaction effect between innovation climate and meaning in life is consistent with the theoretical expectation of the “protection-protection” synergistic effect model proposed by Liu et al. (2018), that is, external environmental resources and internal psychological resources may reinforce rather than substitute for each other. Currently, institutions focus on constructing innovation infrastructure (e.g., maker studios, competition platforms, fault-tolerant policies) should adopt a coordinated rather than isolated investment strategy. It is recommended that institutions accurately match innovation resources to students with different levels of meaning in life development after assessing the characteristics of students’ meaning in life, so as to amplify the enabling effect of the two types of resources on students’ innovative behaviors, avoid the equalization of resource investment, and maximize the innovative output of talent cultivation in vocational universities under limited conditions.

### 6.1 Limitations and future research

Despite achieving the objectives of this study, its research design has five limitations. These limitations may serve as a basis for future research. First, the present study relies entirely on self-reported questionnaire data collected from vocational university students, which renders the findings vulnerable to same-source common method bias. Future research may adopt multi-source data collection approaches that integrate students’ self-assessments, teachers’ evaluations of their daily innovative practices, and institutional administrative records to mitigate measurement bias originating from single-source data. Second, the sample of this study is limited in both geographical coverage and institutional diversity. Participants were recruited solely from three vocational institutions in Guangdong Province, China, representing a narrow range of vocational school types. Subsequent research may employ cross-regional and cross-cultural comparative sampling to examine the invariance of the proposed moderated sequential mediation model across diverse populations and institutional contexts. Third, the current analytical framework fails to disentangle individual subjective perceptions from the school-level construct of overall innovation climate. All variables were measured at the individual student level without hierarchical linear modeling to partition within-school and between-school variances. Future investigations should adopt multi-level analyses with sufficient sample sizes at both the student and institutional levels to unpack these effects and test cross-level interactions. Fourth, cross-sectional data were utilized to test all hypotheses in this work. Although a sequential mediation and moderated mediation model was constructed, the cross-sectional design cannot establish causal directions among variables. Notably, per Bandura’s (1986) triadic reciprocal determinism, mutual interconnections may exist among individuals’ psychological states, environmental conditions, and behavioral outcomes, rather than a unidirectional causal chain running from environment to psychology and then to behavior. The cross-sectional dataset adopted herein is incapable of capturing such dynamic bidirectional causal cycles. Longitudinal tracking studies and experimental manipulations are required in future research to clarify temporal sequences and validate causal pathways. Fifth, only career calling and learning engagement were incorporated as mediating variables to address the core research questions. The results reveal that these two constructs exert partial mediating effects linking innovation climate to innovative behavior. Follow-up studies may introduce additional constructs such as self-efficacy and psychological safety to develop more comprehensive multiple mediation models.

## 7 Conclusion

Based on cross-sectional survey data from 1,789 Guangdong vocational university students, this study constructed a moderated sequential mediation model to unpack the associative pathways linking school innovation climate to students’ innovative behavior. Results verified two independent mediating paths (via career calling and learning engagement) and one sequential chain mediating path, which together accounted for 41.08% of the total indirect association. Meaning in life positively moderated all three core correlational paths, serving as an internal cognitive amplifier that reinforces the positive associations of campus innovation climate with student outcomes. The present study expands vocational innovation research by integrating five key psychological and environmental constructs and empirically validates the synergistic interaction between external campus support and internal meaning in life resources.

## Data Availability

The raw data supporting the conclusions of this article will be made available by the authors, without undue reservation.
