# Supplementary material for: Associations between the innovation climate in vocational universities and students’ innovative behavior: a moderated chain mediation model
Source: Front Psychol. 2026 Jul 17;17:1868146. doi: 10.3389/fpsyg.2026.1868146 (PMC13425911; doi:10.3389/fpsyg.2026.1868146)
Supplement: Supplementary file 1 [file Supplementary_file_1.docx]

**Appendix**

**Innovation Climate Scale**

| No | Item | Strongly disagree | Disagree | Unsure | Agree | Strongly agree |
| --- | --- | --- | --- | --- | --- | --- |
| 1 | During my studies, my classmates demonstrate support and assist each other |  |  |  |  |  |
| 2 | In study, my classmates are willing to share their methods and techniques with each other |  |  |  |  |  |
| 3 | My classmates often communicate and discuss the problems in study and work |  |  |  |  |  |
| 4 | When I have innovative ideas, my classmates actively express their opinions and suggestions |  |  |  |  |  |
| 5 | My teachers respect and tolerate different opinions and objections from students |  |  |  |  |  |
| 6 | My teachers encourage students to make proposals to improve teaching, production, or service |  |  |  |  |  |
| 7 | My teachers will support and assist students to realize their creativity in study and work |  |  |  |  |  |
| 8 | My teacher is a good example of innovation |  |  |  |  |  |
| 9 | Schools advocate for students to try new things and learn from mistakes |  |  |  |  |  |
| 10 | The school appreciates and recognizes students who are innovative and enterprising |  |  |  |  |  |
| 11 | Schools often offer rewards for students’ innovative ideas |  |  |  |  |  |
| 12 | The school advocates freedom, openness, and innovation |  |  |  |  |  |

**Career Calling Scale**

| No | Item | Strongly disagree | Disagree | Unsure | Agree | Strongly agree |
| --- | --- | --- | --- | --- | --- | --- |
| 1 | I aspire to pursue a career that can benefit others. |  |  |  |  |  |
| 2 | The job I want to do is to contribute to society. |  |  |  |  |  |
| 3 | I do not care if my career benefits others or society. |  |  |  |  |  |
| 4 | I want to do something useful to society through my career. |  |  |  |  |  |
| 5 | I feel an invisible force pushing me to pursue a career. |  |  |  |  |  |
| 6 | I am compelled by some force to choose my future career. |  |  |  |  |  |
| 7 | I feel destined to pursue my future career. |  |  |  |  |  |
| 8 | I take one profession for granted compared to others. |  |  |  |  |  |
| 9 | I will find meaning in my profession. |  |  |  |  |  |
| 10 | I want to find a job where I can feel that I am worth something. |  |  |  |  |  |
| 11 | My career is a way to show my value in life. |  |  |  |  |  |

**Learning Engagement Scale**

| No | item | Never | Hardly ever (a few times a year) | Rarely (once a month) | Sometimes (two or three times a month) | Often (once a week) | Very often (several times a week) | Always (every day) |
| --- | --- | --- | --- | --- | --- | --- | --- | --- |
| 1 | I enjoy studying immediately after waking up in the morning |  |  |  |  |  |  |  |
| 2 | When I study, I feel energetic |  |  |  |  |  |  |  |
| 3 | Even if the study is not smooth, I will not be discouraged and can persevere |  |  |  |  |  |  |  |
| 4 | I can keep studying for a long time and do not need breaks in between |  |  |  |  |  |  |  |
| 5 | When studying, I can recover quickly even if I am mentally tired |  |  |  |  |  |  |  |
| 6 | When I study, I am energetic and motivated |  |  |  |  |  |  |  |
| 7 | I find study challenging |  |  |  |  |  |  |  |
| 8 | Learning inspires me |  |  |  |  |  |  |  |
| 9 | I am enthusiastic about learning |  |  |  |  |  |  |  |
| 10 | I am proud of my study |  |  |  |  |  |  |  |
| 11 | I find it purposeful and rewarding |  |  |  |  |  |  |  |
| 12 | While studying, I forget everything around me |  |  |  |  |  |  |  |
| 13 | When I am studying, I feel that time flies |  |  |  |  |  |  |  |
| 14 | When I study, all I think about is studying |  |  |  |  |  |  |  |
| 15 | I cannot put down what I am studying |  |  |  |  |  |  |  |
| 16 | I am immersed in my studies |  |  |  |  |  |  |  |
| 17 | I feel happy when I devote myself to my studies |  |  |  |  |  |  |  |

**Innovative Behavior Scale**

| No | Item | Strongly disagree | Disagree | Unsure | Agree | Strongly agree |
| --- | --- | --- | --- | --- | --- | --- |
| 1 | I am always looking for opportunities to improve learning and working methods as well as processes |  |  |  |  |  |
| 2 | I often try to adopt new methods to solve the problems in my study and work |  |  |  |  |  |
| 3 | I often think about things from different angles |  |  |  |  |  |
| 4 | I never miss an opportunity to learn and discover a problem |  |  |  |  |  |
| 5 | I will always advise others to adopt new methods of learning or working |  |  |  |  |  |
| 6 | I often take risks to support innovative ideas or innovative ideas |  |  |  |  |  |
| 7 | I will often introduce some new learning and working methods to my classmates |  |  |  |  |  |
| 8 | I always test the effectiveness of new methods of study and work |  |  |  |  |  |

**Meaning in Life Scale**

| No | Item | Completely disagree | Strongly disagree | Disagree | Unsure | Agree | Strongly agree | Completely agree |
| --- | --- | --- | --- | --- | --- | --- | --- | --- |
| 1 | I know exactly what gives my life meaning |  |  |  |  |  |  |  |
| 2 | I do not have a noticeably clear purpose in life |  |  |  |  |  |  |  |
| 3 | I have a noticeably clear sense of purpose in my life |  |  |  |  |  |  |  |
| 4 | I know what my life means |  |  |  |  |  |  |  |
| 5 | I have found a satisfying purpose in life |  |  |  |  |  |  |  |
